# Inhibition of protein farnesylation enhances the chemotherapeutic efficacy of the novel geranylgeranyltransferase inhibitor BAL9611 in human colon cancer cells

**DOI:** 10.1054/bjoc.2001.1820

**Published:** 2001-06

**Authors:** A Di Paolo, R Danesi, S Caputo, M Macchia, M Lastella, U Boggi, F Mosca, A Marchetti, M Del Tacca

**Affiliations:** Division of Pharmacology and Chemotherapy, andDivision of General Surgery and Transplants, Department of Oncology, Transplants and Advanced Technologies in Medicine; Department of Pharmaceutical Sciences, University of PisaUniversity of Chieti, Italy

**Keywords:** isoprenylation, colon cancer, ras superfamily, isoprenyl transferases, inhibitors, cytotoxicity

## Abstract

Proteins belonging to the ras superfamily are involved in cell proliferation of normal and neoplastic tissues. To be biologically active, they require post-translational isoprenylation by farnesyl-transferase and geranylgeranyl-transferase. Enzyme inhibition by drugs may thus represent a promising approach to the treatment of cancer. Therefore, the combined effect of BAL9611, a novel inhibitor of geranylgeranylation, and manumycin, a farnesyl-transferase inhibitor, was evaluated on the SW620 human colon cancer cell line which harbours a mutated K-ras gene. BAL9611 and manumycin dose-dependently inhibited SW620 cell growth with 50% inhibitory concentration (IC _50_) of 0.47 ± 0.03 and 5.24 ± 1.41 μM (mean ± SE), respectively. The isobologram analysis performed at the IC _50_ level revealed that the combined treatment was highly synergistic with respect to cell growth inhibition. BAL9611 and manumycin were able to inhibit the geranylgeranylation of p21rhoA and farnesylation of p21ras; both drugs inhibited p42ERK2/MAPK phosphorylation, but their combination was more effective than either drug alone. Moreover, the enhanced inhibition of cell growth in vitro by the BAL9611-manumycin combination was also observed in vivo in CD nu/nu female mice xenografted with SW620 tumours. Finally, both drugs were able to induce cell death by apoptosis in vitro and in vivo, as demonstrated by perinuclear chromatin condensation, cytoplasm budding and nuclear fragmentation, and interoligonucleosomal DNA digestion. In conclusion, the inhibition of protein farnesylation enhances the chemotherapeutic effect of BAL9611 in vitro and in vivo in a synergistic fashion, as a result of the impairment of post-translational isoprenylation of proteins and phosphorylation of p42ERK2/MAPK, whose activation is associated with post-translational geranylgeranylation and farnesylation of p21rhoA and p21ras. © 2001 Cancer ResearchCampaign http://www.bjcancer.com


British Journal of Cancer (2001) **84**(11), 1535–1543
© 2001 Cancer Research Campaign
doi: 10.1054/ bjoc.2001.1820, available online at http://www.idealibrary.com on IDEAL®

http://www.bjcancer.com

### Inhibition of protein farnesylation enhances the chemotherapeutic efficacy of the novel geranylgeranyltransferase inhibitor BAL9611 in human colon cancer cells

**A Di Paolo[1], R Danesi[1], S Caputo[1], M Macchia[2], M Lastella[1], U Boggi[3], F Mosca[3], A Marchetti[4] and M Del Tacca[1]**

[1]Division of Pharmacology and Chemotherapy, and [3]Division of General Surgery and Transplants, Department of Oncology, Transplants and Advanced Technologies in Medicine; [2]Department of Pharmaceutical Sciences, University of Pisa, [4]University of Chieti, Italy

**Summary** Proteins belonging to the ras superfamily are involved in cell proliferation of normal and neoplastic tissues. To be biologically active, they require post-translational isoprenylation by farnesyl-transferase and geranylgeranyl-transferase. Enzyme inhibition by drugs may thus represent a promising approach to the treatment of cancer. Therefore, the combined effect of BAL9611, a novel inhibitor of geranylgeranylation, and manumycin, a farnesyl-transferase inhibitor, was evaluated on the SW620 human colon cancer cell line which harbours a mutated K-ras gene. BAL9611 and manumycin dose-dependently inhibited SW620 cell growth with 50% inhibitory concentration ($IC_{50}$) of 0.47 ± 0.03 and 5.24 ± 1.41 μM (mean ± SE), respectively. The isobologram analysis performed at the $IC_{50}$ level revealed that the combined treatment was highly synergistic with respect to cell growth inhibition. BAL9611 and manumycin were able to inhibit the geranylgeranylation of p21rhoA and farnesylation of p21ras; both drugs inhibited p42ERK2/MAPK phosphorylation, but their combination was more effective than either drug alone. Moreover, the enhanced inhibition of cell growth in vitro by the BAL9611-manumycin combination was also observed in vivo in CD nu/nu female mice xenografted with SW620 tumours. Finally, both drugs were able to induce cell death by apoptosis in vitro and in vivo, as demonstrated by perinuclear chromatin condensation, cytoplasm budding and nuclear fragmentation, and interoligonucleosomal DNA digestion. In conclusion, the inhibition of protein farnesylation enhances the chemotherapeutic effect of BAL9611 in vitro and in vivo in a synergistic fashion, as a result of the impairment of post-translational isoprenylation of proteins and phosphorylation of p42ERK2/MAPK, whose activation is associated with post-translational geranylgeranylation and farnesylation of p21rhoA and p21ras. © 2001 Cancer Research Campaign http://www.bjcancer.com

**Keywords**: isoprenylation; colon cancer; ras superfamily; isoprenyl transferases; inhibitors; cytotoxicity

Post-translational modification of proteins with isoprenoids was first recognized as a general phenomenon almost two decades ago. In recent years, the understanding of the enzymatic reactions associated with these modifications and their functions in physiological and pathological settings has increased dramatically (Sinensky, 2000). Proteins of the ras superfamily play a pivotal role in a wide variety of cellular functions, including signal transduction, dynamic organization of the cytoskeleton and cell proliferation (Marshall, 1996). Mutational activation of ras genes results in uncontrolled growth and malignant transformation, and ras mutants have been discovered in at least 30% of all human neoplasms (Minamoto et al, 2000). Both wild-type and mutated p21ras proteins require post-translational isoprenylation for proper localization within cellular compartments, and these reactions are catalysed by specific enzymes, the farnesyl-transferase (FTase) (Sinensky, 2000). The development of inhibitors of ras farnesylation was a logical consequence of biological research and has been translated in the clinical setting for the treatment of human malignancies (Rowinsky et al, 1999; Zujewski et al, 2000). However, the model of cancerogenesis due to deregulated ras-dependent pathway has increased its complexity in recent years, because of the discovery of multiple ras-related signalling pathways with different downstream effectors that control cell proliferation, differentiation and apoptosis (Campbell et al, 1998). Among these pathways are those that connect ras to members of rho family of GTPase proteins, which are regulators of actin organization and cell cycle progression (Zohn et al, 1998; Kaibuchi et al, 1999). A number of experimental observations suggested that rho and rac proteins are necessary for cell transformation by oncogenic ras proteins (Olson et al, 1995, 1998; Prendergast et al, 1995), because some characteristics of malignant phenotype seem to be dependent on deregulation of rho and rac functions (Zohn et al, 1998). For these reasons, the use of FTase inhibitors may not be fully effective to control cell proliferation, because geranylgeranylated proteins, including rho, rac and CDC42, play a pivotal role in G1–S-phase transition of cells committed to mitosis (Olson et al, 1995; Lebowitz and Prendergast, 1998). Indeed, the treatment with inhibitors of the enzymes geranylgeranyl-transferase (GGTase) I and II causes cells to arrest at the G1–S boundary (Miquel et al, 1997) and suppresses cyclin-dependent kinase (cdk) activity (Sun et al, 1999). Of note, p21rhoA is involved in G protein-coupled receptor signal transduction (Seasholtz et al, 1999; Sah et al, 2000), and the cellular response to selected growth factors,

*Received 31 July 2000*
*Revised 23 February 2001*
*Accepted 26 February 2001*

*Correspondence to:* M Del Tacca including platelet-derived growth factor, requires protein geranylgeranylation rather than farnesylation (McGuire et al, 1996). Finally, cellular proteins, including K-rasB, may be activated through geranylgeranylation, if the FTase activity is impaired, thus reducing treatment efficacy of FTase inhibitors (Vogt et al, 1996). On these premises, it appears that targeting isoprenylation as a means of controlling cancer cell growth requires the inhibition of both GGTase and FTase for optimal activity.

The inhibition of GGTase may be obtained by compounds that mimic the isoprenylation consensus sequence at the carboxylic terminal of proteins (Vogt et al, 1996; Sun et al, 1998). However, novel GGTase inhibitors have been recently obtained, chemically derived from geranylgeranyl diphosphate (GGPP), in which the biologically labile diphosphate moiety of GGPP, the natural substrate of GGTase, has been replaced by stable isosters (Macchia et al, 1996, 1997). Therefore, the aim of the present study was to investigate the effect of BAL9611, a synthetic analogue of GGPP with potent inhibitory activity on protein geranylgeranylation, on signal transduction through ras proteins and proliferation of the human cancer cell line SW620 in vitro and in vivo, and whether the combined treatment with manumycin, a specific FTase inhibitor, enhances the chemotherapeutic activity of BAL9611.

### MATERIALS AND METHODS

### Drugs and chemicals

Antipain, leupeptin, aprotinin, SDS and proteinase K were purchased from Boehringer Mannheim GmbH (Mannheim, Germany); cell culture media were from HyClone (Cramlington, UK); agarose and 180 bp DNA ladder were purchased from Gibco (Gaithersburg, MD, USA) and acrylamide was from International Biotechnologies Inc (New Haven, CT, USA). DEA and Nonidet P-40 were obtained from ICN Biomedicals Inc (Costa Mesa, CA, USA); mouse IgG1 anti-pan21*ras* monoclonal antibody was purchased from Transduction Laboratories (Lexington, KY, USA), anti-p21rhoA and anti-p42ERK2/MAPK rabbit polyclonal antibodies were from Santa Cruz Biotechnology Inc (Santa Cruz, CA, USA). Horseradish peroxidase-conjugated secondary antibodies and reagents for chemiluminescence detection of proteins in immunoblots (ECL Western detection system) were purchased from Amersham Life Science (Little Chalfont, UK). Bisbenzimide trihydrochloride (Hoechst 33258) was from Sigma as well as all other chemicals (St Louis, MO, USA).

The tripotassium salt of (E,E,E)-1-ethyl-1-[(4,8,12,16-tetramethyl-3,7,11,15-heptadecatetraenyl)hydroxyphosphoryl]-propylphosphonic acid (BAL9611, Figure 1) was synthesized at the department of Pharmaceutical Sciences, University of Pisa (Macchia et al, 1996). The drug was dissolved in water/ethanol (50:50, v/v) at 10 mM and stored at –20˚C. Manumycin (Figure 1) was kindly provided by Dr M Hara, Kyowa Hakko Kogyo Co (Tokyo, Japan); the drug was dissolved in sterile distilled water containing DMSO 0.05% at 10 mM concentration, and stored at –20˚C. Manumycin and BAL9611 were diluted in sterile culture medium immediately before their use.

### Cell culture

The human colorectal cancer cell line SW620 was obtained from the American Type Culture Collection (Rockville, MD, USA).

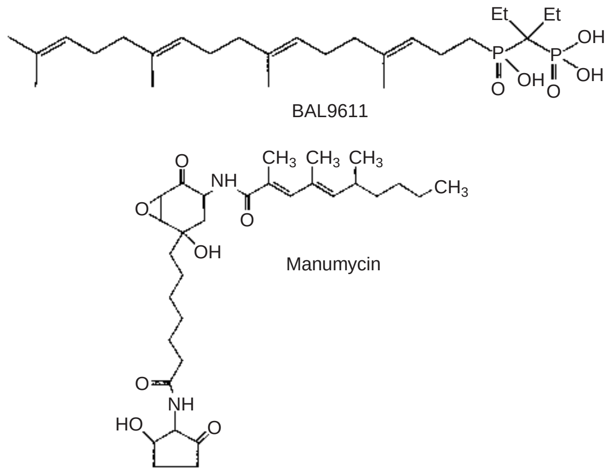

**Figure 1** Chemical structure of BAL9611 and manumycin

SW620 cells were maintained in RPMI 1640 medium, supplemented with 10% FBS, penicillin (50 IU $ml^{-1}$), streptomycin (50 µg $ml^{-1}$) and L-glutamine (2 mM). Cells were grown in 75 $cm^2$ tissue culture flasks (Nunc, Roskilde, Denmark) and kept in a humidified atmosphere of 5% $CO_2$ at 37˚C. Cells were harvested with 2 mM EDTA when they were in log phase of growth, and maintained at the above-described culture conditions for all experiments.

### PCR analysis of K-*ras* sequence in SW620 cell line

The analysis of the codon 12 of K-*ras* gene was performed in SW620 cells by oligodeoxynucleotide hybridization, as previously described (Marchetti et al, 1997). The primers used to amplify the K-*ras* gene upstream and downstream codon 12 were as follows: 5′-GGCCTGCTGAAAATGACTGA-3′ and 5′-TGATTCTGAATTAGCTGTAT-3′. The PCR reaction was performed as follows: initial denaturation, 4 min at 94˚C; amplification, 30 s at 94˚C, 30 s at 54˚C and 1 min at 72˚C for 35 cycles; elongation, 10 min at 72˚C. The amplified products of the PCR reaction were denatured, blotted onto nylon membranes and then hybridized with $^{32}$P-labelled oligonucleotide probes specifically designed to detect ras mutations (Marchetti et al, 1997).

### Immunoblot analysis of p21ras farnesylation, p21rhoA geranylgeranylation and p42ERK2/MAPK phosphorylation

SW620 cells were exposed to BAL9611 0.01–1 µM or manumycin 1 µM for 72 h to document their effect on p21rhoA geranylgeranylation, while p21ras farnesylation was evaluated in cells treated with manumycin 1–10 µM or BAL9611 at 0.1 µM for 72 h. Cells exposed to drugs and untreated controls were harvested with 2 mM EDTA and washed thrice with phosphate-buffered saline (PBS), pH 7.4, and finally solubilized at 4˚C for 45 min in Tris-base 50 mM, pH 7.6, 2 mM EDTA, 100 mM NaCl, 1% (v/v) Nonidet P-40, 1 mM phenylmethylsulfonyl fluoride (PMSF) and 2 µg $ml^{-1}$ each of aprotinin, pepstatin and antipain and the protein phosphatase inhibitors sodium metavanadate and sodium fluoride (200 µM each). Cell lysate was then centrifuged at 15 000 rpm for 20 min, and the pellet was discarded. Aliquots of supernatants were used to measure protein concentration, and the remaining

 *© 2001 Cancer Research Campaign*

samples were used for immunoblot analysis. Samples (40 μg each) were boiled for 5 min in SDS-sample buffer (50 mM Tris-base pH 6.8, 2% sodium dodecyl sulfate (SDS), 100 mM dithiothreitol, 10% glycerol and 0.025% β-mercaptoethanol) and separated on 15% SDS-polyacrylamide gel electrophoresis (SDS-PAGE). Blots were probed with anti-p21ras or anti p21rhoA antibodies (1:500 and 1:1000, respectively), and detected with the use of horseradish peroxidase-conjugated secondary antibody (dilution, 1:1000) (Danesi et al, 1995, 1996). The membranes were then exposed to a Kodak X-Omat AR film, and optical densities of films obtained in nine independent experiments were quantified as described below in Analysis of data. The effect of drugs on p42ERK2/MAPK phosphorylation was evaluated in SW620 cells treated with BAL9611 0.01–1 μM, manymycin 1 μM and with BAL9611 0.01 μM plus manumycin 0.1 μM for 72 h. Cells were harvested and solubilized as reported above; cellular lysates were centrifuged for 20 min at 15 000 rpm, and aliquots of supernatants were separated to measure protein concentration. Equivalent amounts of cellular proteins (40 μg) were separated on 15% SDS-PAGE (Danesi et al, 1995, 1996), transferred onto Immobilon-P membrane, probed with anti-p42ERK2/MAPK antibody (dilution, 1:1000), and detected as described above. Optical densities of films obtained in nine independent experiments were quantified as described below in Analysis of data.

### Assay of apoptosis by BAL9611 and manumycin

To demonstrate whether BAL9611 and manumycin were able to trigger apoptosis, SW620 cells were treated with BAL9611 0.01–1 μM or manumycin 1 μM for 72 h. Cells were harvested with EDTA 2 mM, washed thrice with PBS, pH 7.4, and of $3 \times 10^6$ cells for each drug concentration were centrifuged at 2000 rpm for 15 min. Cells were solubilized in TE buffer (1 mM EDTA, 10 mM Tris-base, pH 7.5) containing 0.2% (v/v) Triton X-100 for 90 min at 4˚C. Samples were centrifuged at 15 000 rpm for 1 h at 4˚C and clear supernatants containing fragmented chromatin were incubated at 42˚C for 30 min with proteinase K (200 μg $ml^{-1}$) and then diluted 1:1 in phenol:chloroform:isoamyl alcohol, vigorously shaken for 30 s, and centrifuged at 15 000 rpm for 10 min. Supernatants were again collected and mixed with 100 μl of NaCl 5 M, 1 ml of cold ethanol, and 1 μl of glycogen. The suspensions were kept at –20˚C overnight to precipitate DNA fragments and then centrifuged at 15 000 rpm for 30 min; the supernatants were discarded and the pellets were washed with 70% ethanol and dried under air flow.

Each sample was resuspended in TE buffer (1 mM EDTA, 10 mM Tris-base, pH 7.5), containing 1 mg $ml^{-1}$ boiled bovine pancreatic RNAse A, incubated at 40˚C for 60 min, and mixed with DNA sample buffer (15 mM EDTA pH 8.0, 0.1% SDS, 0.025% xylene cyanole, 0.025% bromophenol blue and 0.5% glycerol). Separation of DNA fragments was obtained by electrophoresis in 1% agarose gel, in TAE buffer (Tris-base 32 mM, 1% glacial acetic acid and 1 mM EDTA); bands were visualized by ethidium bromide staining under UV light and the gels were photographed with a Polaroid MP4 Land camera (Polaroid, Cambridge, MA, USA).

Moreover, the induction of apoptosis by BAL9611 and manumycin in SW620 cells was further demonstrated by staining with bisbenzimide trihydrochloride (Hsueh et al, 2000). Briefly, cells were harvested by trypsin-EDTA, washed twice in PBS, fixed in 3% paraformaldheyde and incubated at room temperature for 10 min. Then cells were washed with PBS, resuspended in PBS containing 8 μg $ml^{-1}$ of bisbenzimide trihydrochloride and incubated for 15 min at room temperature. Aliquots of cell suspension were placed on sylanized microscope glass slides and cells were counted and scored for incidence of apoptotic features (i.e., chromatin condensation, nucleus fragmentation) using a Leica microscope (Leica, Germany). The apoptotic index (AI) was calculated as the percentage ratio between the number of cells displaying apoptotic features and the total number of counted cells.

### Analysis of cytotoxicity and drug interaction

To evaluate the effect of BAL9611 and manumycin alone and in combination on SW620 cell growth, as well as the type of interaction with respect to cytotoxicity, SW620 cells were seeded at $1.5 \times 10^6$ cells $well^{-1}$ in 60-mm sterile dishes for cell culture, in a final volume of 3 ml $well^{-1}$, and incubated at 37˚C in 5% $CO_2$. After 48 hours, cells were treated with BAL9611 0.01–10 μM and manumycin 0.01–25 μM alone or in combination for 72 h; for comparison, separate cell cultures were given 5-fluorouracil (5-FU) 0.01–100 μM. At the end of experiment, cultures were harvested with 2 mM EDTA and the number of surviving cells was counted by haemocytometer. The inhibitory concentration of BAL9611 and manumycin on cell growth at the 50% level ($IC_{50}$) as well as the type of drug interaction were assessed in nine independent experiments as described in Analysis of data.

### In vivo studies

The chemotherapeutic effect of BAL9611 and manumycin in vivo was investigated in athymic mice. Briefly, SW620 cells in log-phase of growth, maintained at the above-mentioned culture conditions, were harvested by scraping and $10^7$ cells were resuspended in serum-free RPMI-1640 culture medium. Cells were injected subcutaneously between the scapulae of 7-week-old female CD nu/nu mice (Harlan Nossan, Comerio, Italy). Mice were housed in laminar-air-flow caging system (Techniplast, Buguggiate, Italy) and provided with sterilized water, food and bedding ad libitum. Tumour masses became detectable 3 days after cell inoculum and mice were randomly divided in 4 groups of 6 animals each. Mice were given i.p. BAL9611 10 mg $kg^{-1}$ and manumycin 8 mg $kg^{-1}$, alone or in combination, or vehicle (control group), daily for 5 days. The dose of BAL9611 was chosen on the basis of preliminary experiments aimed at defining a treatment schedule with significant antitumor activity and tolerable toxicity, while manumycin dosing was selected on the basis of a previous report (Hara et al, 1993). Tumour growth was evaluated daily, by measuring the size of each mass, and its volume (V) was calculated as follows: $V = 1/2 \times A \times B^2$, where A and B are the tumour length and width (in mm), respectively (Tomayko and Reynolds, 1989). Tumour growth was monitored for further 3 weeks after the end of treatment, after which mice were sacrified, and tumours were explanted and measured.

In order to demonstrate the induction of apoptosis by drugs in xenografts, H&E-stained section of tissue specimens were examined using light microscopy for morphological evidence of apoptosis. Briefly, following the above described experimental conditions, 16 female CD nu/nu mice were injected with $10^7$ SW620 cells/mouse between scapulae, and treatment started when tumour mass became palpable. Mice were treated at the same dosages described above for 5 consecutive days (4 mice/group),

© 2001 Cancer Research Campaign and at the end of treatment xenografts were excised, embedded in paraffin, and 3-μm sections were obtained. Specimens were stained with H&E and cells were counted and scored for the occurrence of morphological evidence of apoptosis (Gandhi et al, 1998). Again, the apoptotic index (AI) was calculated as the percentage ratio between the number of cells displaying apoptotic features and the total number of cells counted.

### Analysis of data

The effects of BAL9611 and manumycin on protein isoprenylation as well as on kinase phosphorylation were assessed by capturing the images corresponding to the non-isoprenylated (p21ras and p21rhoA) or phosphorylated (p42ERK2/MAPK) protein bands in the immunoblotting (upper position) and isoprenylated or non-phosphorylated protein bands (lower position in the immunoblots). Film densities of protein blots were examined with the Kontron Imaging analysis system KS300 (Kontron Elektronic, Eching, Germany) connected to a TK-1280E colour video camera (JVC, Tokyo, Japan), and expressed as mean percentage ± standard error (SE) of arbitrary units of grey values with respect to controls (Di Paolo et al, 2000). In order to compensate for the decrease in protein synthesis as a consequence of treatment of cells with test compounds, the inhibitory effect of BAL9611 and manumycin on p21ras and p21rhoA isoprenylation as well as on p42ERK2/MAPK phosphorylation was expressed as the ratio of isoprenylated/phosphorylated versus non-isoprenylated/non-phosphorylated bands. The non-linear least squares curve fitting of the experimental data of protein immunoblots (ratios of modified vs. unmodified proteins) allowed the calculation of the drug concentration producing a 50% decrease ($IC_{50}$) in the isoprenylation or phosphorylation of target proteins. The proliferation of treated cells was expressed as the mean percent values ± SE of cell numbers counted at the end of the experiment with respect to untreated controls. Experimental data were modelled by non-linear least squares curve fitting in order to obtain the drug concentration that produced a 50% inhibition ($IC_{50}$) of SW620 cell proliferation. In addition to this, the type of drug interaction in the combined treatment was analysed at the 50% isoeffect level by using the isobologram method previously described (Steel and Peckham, 1979; Greco et al, 1995). Briefly, 3 isoeffect curves (isobolograms) were constructed: mode I in the case of independent mechanism of drug action (heteroaddition), and mode IIa and IIb if BAL9611 and manumycin were acting by a similar mechanism (isoaddition). Regardless of the mechanism of action of drugs, the combination was considered to be additive if data points of the drug combination are included in the area surrounded by 3 lines (envelope of additivity). On the contrary, if experimental data fall to the left or to the right of the envelope, the combined effect of 2 drugs is greater (supra-additive, synergism) or lower (sub-additive) than the sum of single drug effects, respectively. Finally, if point coordinates are greater than $IC_{50}$ values of one or both drugs, the combined effect is lower than the effect of a single pharmacological agent (protection). Both sub-additive and protective effects were considered to be antagonistic.

All results are obtained in triplicate sets of experiments, each done in triplicate and results are given as mean values ± SE. The two-tail unpaired Student's *t*-test or ANOVA followed by the Student–Newmann–Keuls test were used to assess statistical differences of data obtained in control and treated cells with respect to immunoblotting, apoptosis and in vitro and in vivo cytotoxicity studies. *P* values lower than 0.05 were considered significant.

### RESULTS

### K-ras gene mutation in SW620 cells

Mutated K-*ras* oncogenes are frequently found in colon cancers, and for this reason the analysis of K-*ras* gene sequence in SW620 cell line was performed. Indeed, SW620 tumour cells displayed a GGT (Gly) → GTT (Val) point mutation at codon 12 of one allele, as shown by dot blot analysis with mutation-specific probes.

### Inhibition of p21rhoA geranylgeranylation, p21ras farnesylation and p42ERK2/MAPK phosphorylation by BAL9611 and manumycin

Western blot analysis showed that BAL9611 and manumycin inhibited the geranylgeranylation and farnesylation of p21rhoA (Figure 2) and p21 ras (Figure 3), respectively. In particular, BAL9611 reduced dose-dependently the optical density of the lower protein band of each lane, corresponding to the geranylgeranylated p21rhoA (Figure 2A), with significant effect starting from 0.1 μM (Figure 2B). Although some reduction in the bands of p21rhoA was observed while increasing the concentration of BAL9611 in the culture medium, image analysis demonstrated that the geranylgeranylated protein was reduced more than the non-geranylgeranylated p21rhoA, thus resulting in a 'true' dose-dependent impairment of protein geranylgeranylation (Figure 2B), not influenced by variations of protein synthesis in cells treated with BAL9611. The calculation of the $IC_{50}$ by fitting the optical density ratios provided a value of 0.21 ± 0.03 μM. On the contrary, the geranylgeranylation of p21rhoA was unaffected by manumycin 1 μM for 72 h (Figure 2A and 2B), a drug concentration that was indeed able to inhibit p21ras farnesylation (Figure 3). Manumycin dose-dependently inhibited the isoprenylation of p21ras, as demonstrated by the immunoblots showing a progressive

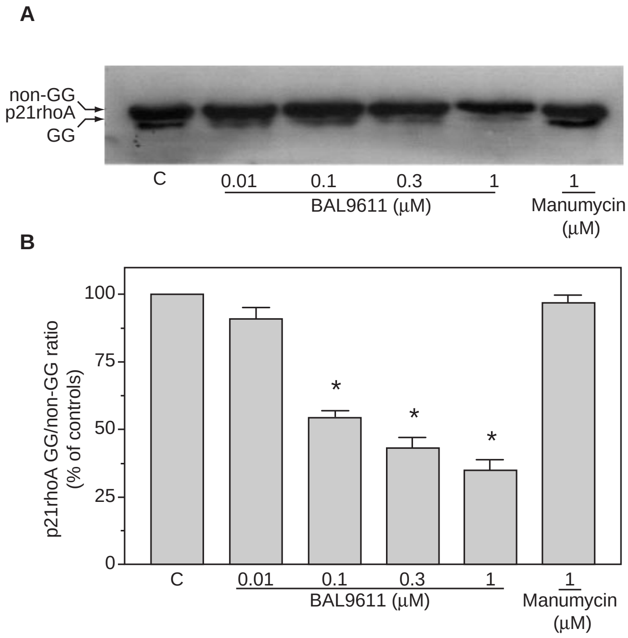

**Figure 2** Western blot analysis (**A**) of p21rhoA from cells treated with BAL9611 0.01–1 μM or manumycin 1 μM for 72 h and (**B**) image analysis of geranylgeranylated (GG) vs. non-GG isoforms of p21rhoA. Columns: mean values; vertical bars: S.E.; *$P < 0.05$ vs. control (C) values

*© 2001 Cancer Research Campaign*

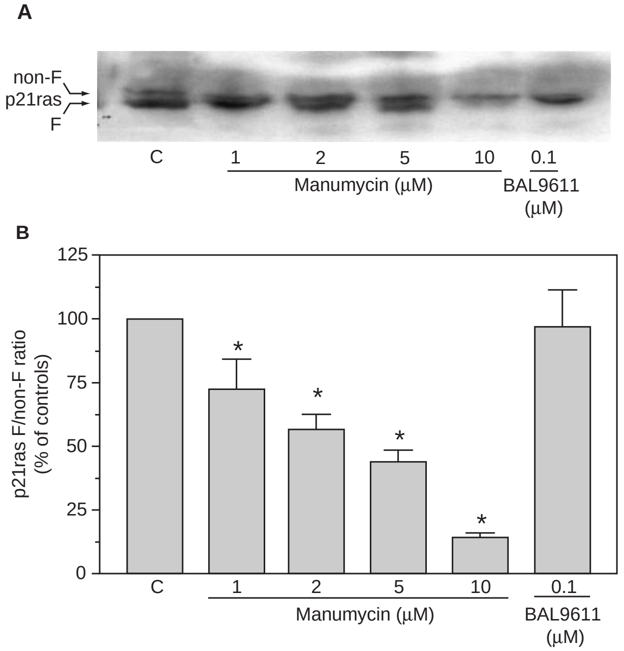

**Figure 3** Western blot analysis (**A**) of p21ras from cells treated with manumycin 1–10 μM or BAL9611 0.1 μM for 72 h and (**B**) image analysis of farnesylated (F) vs. non-F isoforms of p21ras. Columns: mean values; vertical bars: S.E.; *$P < 0.05$ vs. control (C) values

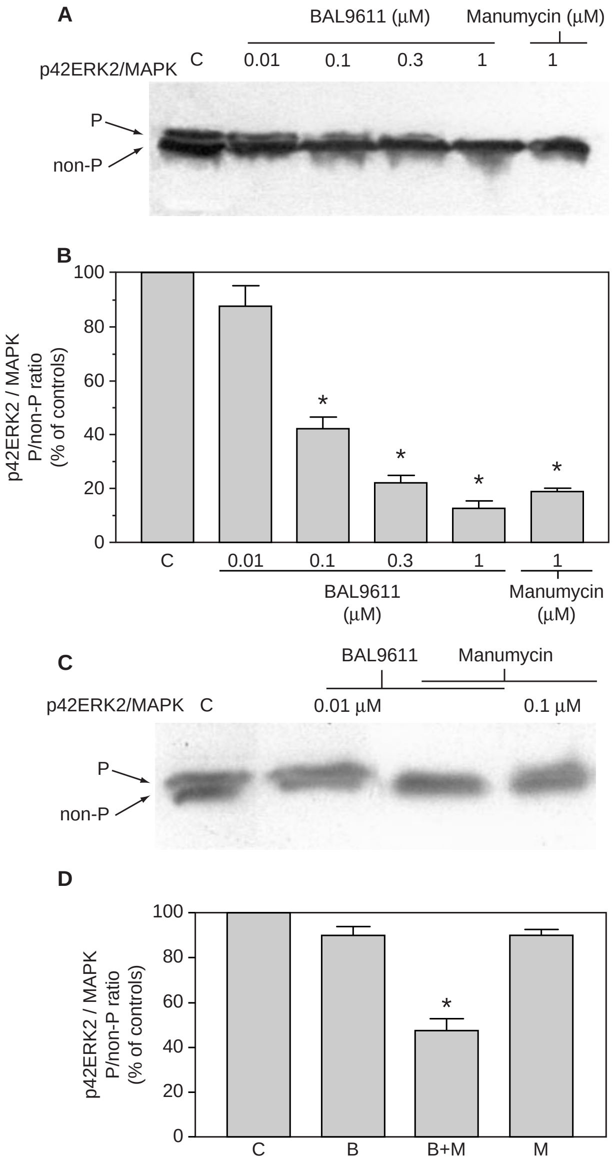

**Figure 4** Western blot analysis (**A**) of p42ERK2/MAPK from cells treated with BAL9611 0.01–1 μM or manumycin 1 μM for 72 h and (**B**) image analysis of phosphorylated (P) vs. non-P isoforms of p42ERK2/MAPK. Columns: mean values; vertical bars: S.E.; *$P < 0.05$ vs. control (C) values. Effect of combined treatment with BAL9611 and manumycin on p42ERK2/MAPK phosphorylation: Western blot analysis (**C**) and image analysis (**D**) of control cells (C), and cells treated with BAL9611 0.01 μM (B), manumycin 0.1 μM (M) or combination (B+M). Columns: mean values; vertical bars: S.E.; *$P < 0.05$ vs. control (C) values enrichment in the upper band of non-farnesylated peptide (Figure 3A), whereas BAL9611 0.1 μM did not impair the farnesylation of p21ras (Figure 3A and B). As also observed for BAL9611, the ratio between the 2 protein bands that were detected in p21ras immunoblots indicated that the inhibition of farnesylation was not related to the reduction in p21ras abundance in cancer cells as a result of drug treatment (Figure 3A and B), and the calculated $IC_{50}$ value of manumycin was 2.99 ± 0.59 μM. Finally, the phosphorylation of p42ERK2/MAPK was reduced dose-dependently by BAL9611 and manumycin (Figure 4A), and the decrease was confirmed by the assessment of the ration between the phosphorylated and non-phosphorylated protein bands (Figure 4B). At the concentration of 0.01 μM BAL9611 and 0.1 μM manumycin, the relative amount of phosphorylated p42ERK2/MAPK was inhibited by 9.83 ± 3.55% and 2.29 ± 0.42% (Figure 4C and D), respectively, as compared to untreated controls, whereas the combined treatment at the same drug concentrations reduced the phosphorylation of p42ERK2/MAPK by 52.68 ± 5.47% (Figure 4C and 4D).

### BAL9611 and manumycin induce apoptosis in SW620 cells

The occurrence of apoptosis was demonstrated by the recovery of small fragmented DNA from the cytoplasm of cells treated with isoprenylation inhibitors. The analysis by agarose gel electrophoresis showed the presence of a distinctive DNA laddering with fragments of multiple of the internucleosomal length of 180 bp after treatment with BAL9611 0.01–1 μM or manumycin 1 μM (Figure 5A). These results were confirmed by the assessment of the AI in SW620 cells exposed to the same concentrations of BAL9611 and manumycin. In treated cells the staining with the compound Hoechst 33258 ensured the detection of early and late phases of apoptosis. In fact, the AI was significant higher in cells treated with BAL9611 0.1 to 1 μM or manumycin 1 μM than in controls (Figure 5B), and the induction of apoptosis was dose-dependent in the case of BAL9611. In agreement with the in vitro data, results obtained in SW620 xenografts revealed that both drugs were able to induce apoptosis, due to the presence of perinuclear condensation of chromatin, cytoplasmic budding, cell shrinkage and, finally, formation of apoptotic bodies, as observed in H&E-stained sections. The AI was significant higher in xenografts treated with BAL9611 plus manumycin with respect to controls, and the combined treatment was superior to single agents (Figure 5B). Finally, BAL9611 was more effective in inducing apoptosis in xenotransplanted SW620 cells than manumycin (AI values, 13.5 ± 1.2 and 11.3 ± 0.9, respectively), and it was in agreement with the

*© 2001 Cancer Research Campaign*

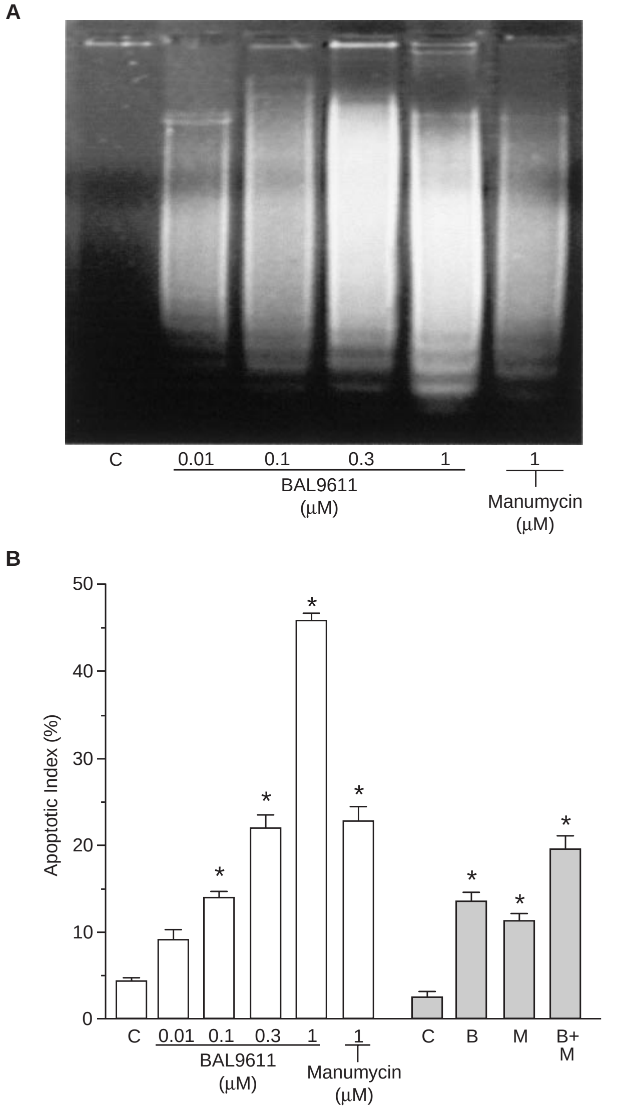

**Figure 5** Agarose gel electrophoresis (**A**) of DNA from cells exposed to BAL9611 0.01–1 μM or manumycin 1 μM, in which the occurrence of apoptotic death is evidenced by DNA laddering. (**B**) Calculation of apoptotic index (AI) in vitro (open columns) and in vivo (grey columns), and dose-dependent induction of apoptosis by exposure of SW620 cells to increasing concentrations of BAL9611 in vitro. *, $P < 0.05$ vs. controls (C); B, BAL9611 10 mg/kg/die i.p.; M, manumycin 8 mg/kg/die i.p.; B+M, combination of drugs lower $IC_{50}$ value of the GGTase inhibitor with respect to manumycin.

### Synergistic inhibition of SW620 cell proliferation by BAL9611 and manumycin

BAL9611, manumycin and 5-FU reduced SW620 cell proliferation in a dose-dependent manner (Figure 6), and the $IC_{50}$ values were 0.49 ± 0.03 μM, 2.73 ± 0.38 μM and 5.24 ± 1.41 μM, respectively. The isobologram analysis of drug interaction between BAL9611 and manumycin demonstrated that BAL9611 0.01–10 μM in combination with increasing concentrations of manumycin, from 0.1 to 5 μM, shifted the survival curve of SW620 cells toward the left in the semilogarithmic plot (Figure 7). The $IC_{50}$ values of BAL9611 in the combined treatment for increasing concentrations of manumycin from 0.1 to 5 μM were: 0.47 ± 0.11 and 0.19 ± 0.02 μM, 65.0 ± 5.3, 4.2 ± 0.09 and 3.3 ± 0.06 nM, respectively ($P < 0.05$ with respect to BAL9611 alone). The results obtained from the isobologram analysis performed at the 50%

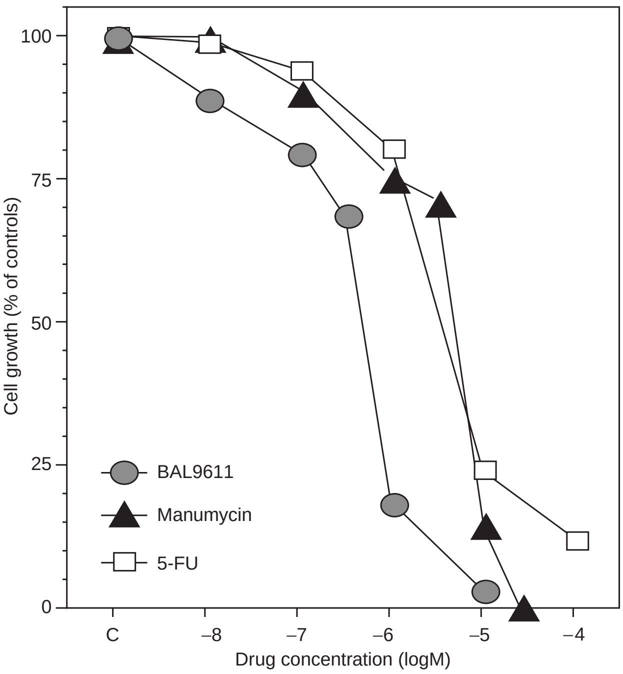

**Figure 6** Dose-dependent inhibition of SW620 cell proliferation by BAL9611, manumycin and 5-FU. Points: mean values; C, controls. All SE values are lower than 5% of the respective mean values

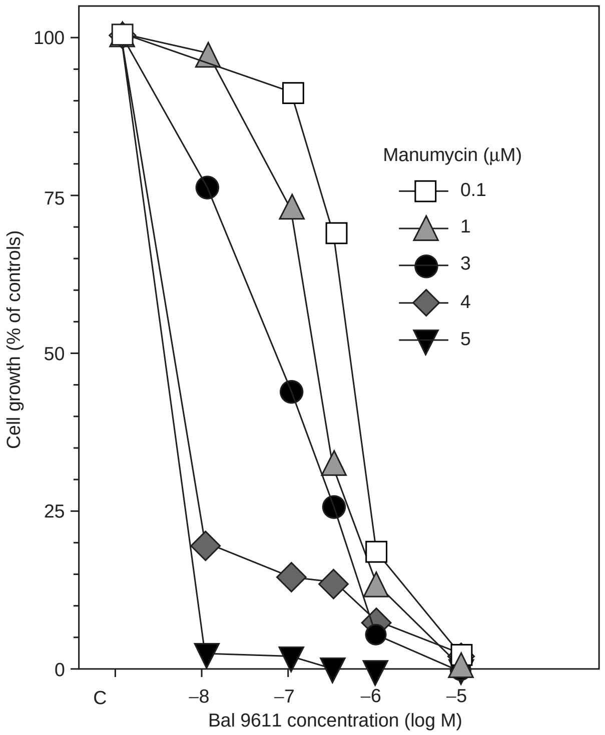

**Figure 7** Dose-dependent enhancement of BAL9611 cytotoxicity by manumycin 0.1–5 μM. Points: mean values; C, controls; SE values are lower than 5% of the respective mean values

*© 2001 Cancer Research Campaign*

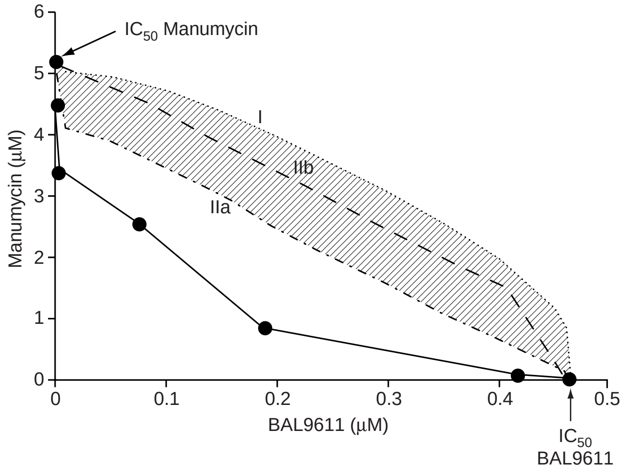

**Figure 8** Isobologram analysis performed at the 50% isoeffect level of BAL9611 plus manumycin. The experimental points corresponding to mean $IC_{50}$ values of drugs lie to the left and below the envelope of additivity (shaded area), indicating synergistic cytotoxic effect on SW620 cells. The points of $IC_{50}$ of BAL9611 and manumycin are shown by arrows isoeffect level demonstrated that the combined treatment of BAL9611 plus manumycin was synergistic, because the experimental $IC_{50}$ values of the combination fell to the left of the envelope of additivity (Figure 8).

### Enhanced growth inhibition of SW620 cells in vivo by BAL9611 and manumycin

SW620 cells injected s.c. in CD nu/nu mice grew rapidly and tumour masses became detectable 3 days after xenotransplantation. Tumours in control animals showed a progressive enlargement in their dimensions, and a mean volume of 900 $mm^3$ was reached at the end of the experimental period (Figure 9). Both BAL9611 and manumycin were able to inhibit tumour growth, and their chemotherapeutic effect was significant starting on the 21st day after implant as compared to controls (Figure 9). In the group of animals receiving the combined treatment with BAL9611 and manumycin, the reduction in tumour growth was significant on day 14 with respect to controls (Figure 9). The tumour growth curve of BAL9611+manumycin showed a moderate increase, divergent from that of controls, as well as from BAL9611– and manumycin-treated animals, and at day 21 and 28 the tumour volume was significantly different from that of controls and of animals given BAL9611 and manumycin alone (Figure 9).

### DISCUSSION

The present study provides evidence that the cytotoxic effect of the geranylgeranyltransferase inhibitor BAL9611 is enhanced in vitro and in vivo on SW620 colon cancer cells by combination with manumycin, an inhibitor of protein farnesylation. These data support the evidence that optimum chemotherapeutic effect of inhibitors of protein isoprenylation is obtained when FTase and GGTase inhibitors are combined, in order to target both types of post-translational modification of proteins. In this study, the synergistic cytotoxic effect on SW620 human colorectal cancer cells by BAL9611 and manumycin was associated with inhibition of isoprenylation of p21ras and p21rhoA proteins, with enhanced

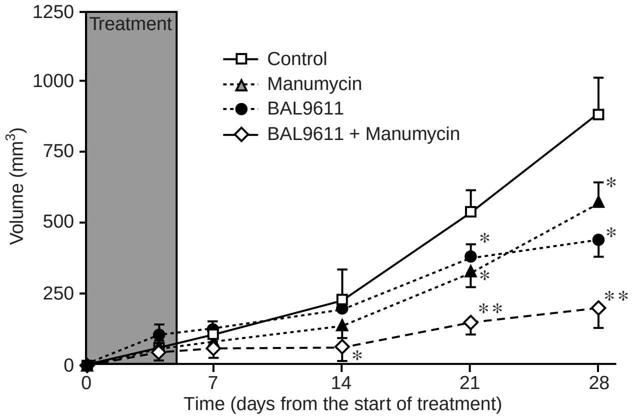

**Figure 9** Chemotherapeutic effect of BAL9611 10 mg/kg/die i.p. and manumycin 8 mg/kg/die i.p. alone or in combination on SW620 tumours xenotransplanted in CD nu/nu mice. Shaded column represents the period of treatment. *, $P < 0.05$ with respect to controls; **, $P < 0.05$ vs. BAL9611 and manumycin alone inhibition of p42ERK2/MAPK phosphorylation. The targeting of ras-dependent signal transduction pathway to inhibit cancer cell proliferation is a promising approach for the therapy of human neoplasms, due to the important role of ras and ras-like proteins in transducing mitogenic signals from cell membrane to the nucleus (Marshall, 1996; Sinensky, 2000). However, due to the large number of proteins which undergo isoprenylation, it is possible that the antiproliferative effect of FTase and GGTase inhibitors could depend also on the interference on all those biochemical pathways which require farnesylated or geranylgeranylated proteins to proceed, as well as proteins involved in cell trafficking, mitotic spindle function, nuclear envelope and signal transduction (Fu and Casey, 1999).

BAL9611 is a novel cell-permeable GGPP analogue endowed with specific inhibitory activity on the GGTase of whole cells (Macchia et al, 1996); this compound is able to impair the post-translational isoprenylation of p21rhoA protein in SW620 cells as well as p42ERK2/MAPK phosphorylation, as demonstrated by the present results. The inhibitory effect of manumycin on p21ras farnesylation and signal transduction through p42ERK2/MAPK pathway has been previously documented in cell lines harbouring wild-type (Di Paolo et al, 2000) or mutated K-*ras* gene (Kainuma et al, 1997). The present evidence that the phosphorylation of p42ERK2/MAPK may be decreased by the GGTase inhibitor BAL9611 suggests that p42ERK2/MAPK activity may be modulated by geranylgeranylated proteins, such as p21rhoA. Therefore, the cooperation between p21ras and p21rhoA appears to be of relevance in the convergent activation of p42ERK2/MAPK, and the use of farnesylation and geranylgeranylation inhibitors appears to be a logical approach for maximum effect on the common kinase pathway located downstream of ras and ras-like proteins. This hypothesis has been confirmed in the present study in SW620 cells exposed to BAL9611 and manumycin, because the combined treatment significantly reduced the phosphorylation of p42ERK2/MAPK with respect to single drugs. The inhibition of both geranylgeranylation and farnesylation of ras proteins by BAL9611 and manumycin induced cell death by apoptosis, as revealed by the typical internucleosomal fragmentation of DNA observed in treated cells and the occurrence of perinuclear chromatin condensation, cytoplasm budding and nuclear fragmentation, as evidenced by in vitro and in vivo microscopic studies. The cytotoxic effect

*© 2001 Cancer Research Campaign*

displayed by BAL9611 in vitro against SW620, a human colorectal cancer cell line harbouring a K-*ras* mutation at codon 12, was superior to that of manumycin. This finding underscores the important role of geranylgeranylated proteins, and of p21rho proteins in particular, in the maintenance of a malignant phenotype by cells harbouring a mutated *ras* gene (Prendergast et al, 1995) and in the promotion of cell proliferation (Hirai et al, 1997; Sun et al, 1998). In addition, the impairment of the function of integrated pathways originating from p21ras and p21rhoA proteins obtained by exposing cells to BAL9611 and manumycin resulted in a marked reduction of tumour growth. The cytotoxic activity of BAL9611 compares well with that of other inhibitors of protein geranylgeranylation, including GGTI-298 (Miquel et al, 1997). The enhanced chemotherapeutic activity of BAL9611 may be dependent on several factors, including effective inhibition of GGTase and improved diffusion through the cell membranes due to the lipophilic structure of BAL9611, as compared to peptidomimetic drugs inhibitors of GGTase, whose in vitro high potency on isolated enzyme is counterbalanced by relatively high doses required in vivo to obtain antitumor effect (Sun et al, 1999). The present study provides the first evidence of the effective inhibition of tumour cell growth by a combination of GGTase and FTase inhibitors containing isoprenoid moieties, since most studies on isoprenylation inhibitors fell short of this experimental evidence. The cytotoxic effect of BAL9611 was synergistically enhanced in vitro by increasing concentrations of manumycin, as confirmed by the shift of the SW620 cell survival curve towards the left, with lower $IC_{50}$ values with respect to single drug treatments. The isobologram analysis demonstrated that the combination was highly synergistic, and this finding confirmed the hypothesis that the cytotoxicity of BAL9611 and manumycin depends on the inhibition of both p21ras and p21rhoA proteins, which are involved in signal transduction, gene expression and cell proliferation (Zohn et al, 1998), by means of integrated pathways, which converge on the downstream effector p42ERK2/MAPK, as also demonstrated by the present results.

The ras proteins expressed in animal cells are encoded by H-ras, N-Ras and K-Ras genes; the latter gene generates p21K-rasA and p21K-rasB for alternative splicing of the fourth exon, which encodes the prenylation consensus sequence; the B isoform is the predominant version produced by this gene (James et al, 1995, 1996). Ras proteins resemble each other closely with the exception of their COOH-terminal tetrapeptides that are designated as CAAX boxes in which C is cysteine, A stands for an aliphatic amino acid, and X is a variable amino acid that dictates the relative specificity of the proteins for the 2 prenyltransferases FTase and GGTase. In particular, the following sequences are specific to ras proteins: CVIM (K-RasB), CIIM (K-RasA), CVVM (N-Ras), and CVLS (H-Ras) (James et al, 1995, 1996). Mutations have been identified in all 3 *ras* genes, in about 30% of all human tumours, including 50% of colon cancers and 90% of pancreatic cancers (Minamoto et al, 2000) and mutations in K-RasB are by far the most frequent in human tumours. Oncogenic K-RasB appears to be farnesylated in transformed cells; however, K-RasB, but not H-Ras, is also a substrate for CAAX GGTase-I, and its affinity for the enzyme is equal to that of p21rap1B, an authentic leucine-terminated substrate for GGTase-I (James et al, 1995, 1996). Therefore, p21K-rasB might become geranylgeranylated when farnesylation is blocked by a farnesyltransferase inhibitor. Indeed, geranylgeranylated p21K-rasB has transforming potential similar to the farnesylated version (James et al, 1996). For these reasons, the exposure of cells to FTase and GGTase is required to block p21K-rasB function, even if the present Western blot assays suggested that in cells exposed to manumycin the possible geranylgeranylation of K-ras was uneffective in order to counteract the cell growth by the FTase inhibitor.

Finally, the administration of BAL9611 and manumycin to nude mice reduced the proliferation of xenotransplanted SW620 cells, in agreement with results obtained in vitro. Although tumours displayed a rather fast regrowth after the discontinuation of single drug administration, this effect was much less evident with the combination treatment; therefore future trials should be designated to achieve a sustained inhibition of isoprenyltransferases by a protracted treatment for enhanced control of tumour growth. In conclusion, the antitumor effect of the geranylgeranylation inhibitor BAL9611 is enhanced by blocking farnesylation and this evidence is based on sound biologic principles; in particular, the data of the present study suggest that isoprenylation of protein should be targeted at both geranylgeranylation and farnesylation for optimal antitumor effect.

### ACKNOWLEDGEMENTS

This work was supported in part by a research grant from Laboratori Baldacci SpA, Pisa, Italy

### REFERENCES

Campbell SL, Khosravi-Far R, Rossman KL, Geoffrey JC and Der CJ (1998) Increasing complexity of Ras signaling. *Oncogene* **17**: 1395–1413

Danesi R, Figg WD, Reed E and Myers CE (1995) Paclitaxel (Taxol®) inhibits protein isoprenylation and induces apoptosis in PC-3 human prostate cancer cells. *Mol Pharmacol* **47**: 1106–1111

Danesi R, Nardini D, Basolo F, Del Tacca M, Samid D and Myers CE (1996) Phenylacetate inhibits protein isoprenylation and growth of the androgen-independent LNCaP prostate cancer cells transfected with the T24 Ha-ras oncogene. *Mol Pharmacol* **49**: 972–979

Di Paolo A, Danesi R, Nardini D, Bocci G, Innocenti F, Fogli S, Barachini S, Marchetti A, Bevilacqua G and Del Tacca M (2000) Manumycin inhibits ras signal transduction pathway and induces apoptosis in COLO320-DM human colon tumour cells. *Br J Cancer* **82**: 905–912

Fu HW and Casey PJ (1999) Enzymology and biology of CaaX protein prenylation. *Recent Prog Horm Res* **54**: 315–342

Gandhi A, Holland PA, Knox WF, Potten CS and Bundred NJ (1998) Evidence of significant apoptosis in poorly differentiated ductal carcinoma in situ of the breast. *Br J Cancer* **78**: 788–794

Greco WR, Bravo G and Parsons JC (1995) The search for synergy: a critical review from a response surface perspective. *Pharmacol Rev* **47**: 331–385

Hara M, Kazuhito A, Akinaga S, Okabe M, Nakano H, Gomez R, Wood D, Uh M and Tamanoi F (1993) Identification of ras farnesyltransferase inhibitors by microbial screening. *Proc Natl Acad Sci USA* **90**: 2281–2285

Hirai A, Nakamura S, Noguchi Y, Yasuda T, Kitagawa M, Tatsuno I, Oeda T, Tahara K, Terano T, Narumiya S, Kohn LD and Saito Y (1997) Geranylgeranylated Rho small GTPases(s) are essential for the degradation of p27Kip1 and facilitate the progression from G1 to S phase in growth-stimulated rat FRTL-5 cells. *J Biol Chem* **272**: 13–16

Hsueh C-T, Chiu C-F, Kelsen DP and Schwartz GK (2000) Selective inhibition of cyclooxygenase-2 enhances mitomycin-C-induced apoptosis. *Cancer Chemother Pharmacol* **45**: 389–396

James GL, Goldstein JL and Brown MS (1995) Polylysine and CVIM sequences of K-RasB dictate specificity of prenylation and confer resistance to benzodiazepine peptidomimetic *in vitro. J Biol Chem* **270**: 6221–6226

James GL, Goldstein JL and Brown MS (1996) Resistance of K-RasB$^{v12}$ proteins to farnesyltransferase inhibitors in Rat1 cells. *Proc Natl Acad Sci USA* **93**: 4454–4458

Kaibuchi K, Kuroda S and Amano M (1999) Regulation of the cytoskeleton and cell adhesion by the rho family GPTases in mammalian cells. *Annu Rev Biochem* **68**: 459–486

 © 2001 Cancer Research Campaign

Kainuma O, Asano T, Hasegawa M, Kenmochi T, Nakagohri T, Tokoro Y and Isono K (1997) Inhibition of growth and invasive activity of human pancreatic cancer cells by a farnesyltransferase inhibitor, manumycin. *Pancreas* **15**: 379–383

Lebowitz PF and Prendergast GC (1998) Non-ras targets of farnesyltransferase inhibitors: focus on rho. *Oncogene* **17**: 1439–1445

Macchia M, Jannitti N, Gervasi G and Danesi R (1996) Geranylgeranyl diphosphate-based inhibitors of post-translational geranylgeranylation of cellular proteins. *J Med Chem* **39**: 1352–1356

Macchia M, Balsamo A, Macchia B, Baldacci M, Danesi R and Del Tacca M (1997) Novel geranylgeranyl-derivatives, process for the preparation thereof and related pharmaceutical compositions. WO 9719091

Marchetti A, Buttitta F, Pellegrini S, Chella A, Bertacca G, Filardo A, Tognoni V, Ferreli F, Signorini E, Angeletti CA and Bevilacqua G (1996) Bronchioloalveolar lung carcinomas: K-ras mutations costant events in the mucinous subtype. *J Pathol* **179**: 254–259

Marshall CJ (1996) Ras effectors. *Curr Opin Cell Biol* **8**: 197–204

McGuire TF, Qian Y, Vogt A, Hamilton AD and Sebti SM (1996) Platelet-derived growth factor receptor tyrosine phosphorylation requires protein geranylgeranylation but not farnesylation. *J Biol Chem* **271**: 27402–27407

Minamoto T, Mai M and Ronai Z (2000) K-ras mutation: early detection in molecular diagnosis and risk assessment of colorectal, pancreas and lung cancers-A review. *Cancer Detect Prev* **24**: 1–12

Miquel K, Pradines A, Sun J, Qian Y, Hamilton AD, Sebti SM and Favre G (1997) GGTI-298 induces G0-G1 block and apoptosis whereas FTI-277 causes G2-M enrichment in A549 cells. *Cancer Res* **57**: 1846–1850

Olson MF, Ashworth A and Hall A (1995) An essential role for rho, rac and cdc42 GPTases in cell cycle progression through G1. *Science* **269**: 1270–1272

Olson MF, Paterson HF and Marshall CJ (1998) Signals from Ras and Rho GTPases interact to regulate expression of p21Waf1/Cip1. *Nature* **394**: 295–299

Prendergast GC, Khosravi-Far R, Solski PA, Kurzawa H, Lebowitz PF and Der CJ (1995) Critical role for Rho in cell transformation by oncogenic Ras. *Oncogene* **10**: 2289–2296

Rowinsky EK, Windle JJ and Von Hoff DD (1999) Ras protein farnesyltransferase: a strategic target for anticancer therapeutic development. *J Clin Oncol* **17**: 3631–3652

Sah VP, Seasholtz TM, Sagi SA and Brown JH (2000). The role of Rho in G protein-coupled receptor signal transduction. *Annu Rev Pharmacol Toxicol* **40**: 459–489

Seasholtz TM, Majumdar M and Heller Brown J (1999) Rho as mediator of G protein-coupled receptor signaling. *Mol Pharmacol* **55**: 949–956

Sinensky M (2000) Recent advances in the study of prenylated proteins. *Biochim Biophys Acta* **1484**: 93–106

Steel GG and Peckham MJ (1979) Exploitable mechanisms in combined radiotherapy-chemotherapy: the concept of additivity. *Int J Radiat Oncol Biol Phys* **5**: 85–91

Sun J, Qian Y, Hamilton AD and Sebti AM (1998) Both farnesyltransferase and geranylgeranyltransferase I inhibitors are required for inhibition of oncogenic K-Ras prenylation but each alone is sufficient to suppress human tumor growth in nude mouse xenografts. *Oncogene* **16**: 1467–1473

Sun J, Blaskovich MA, Knowles D, Qian Y, Ohkanda J, Bailey RD, Hamilton AD and Sebti SM (1999) Antitumor efficacy of a novel class of non-thiol-containing peptidomimetic inhibitors of farnesyltransferase and geranylgeranyltransferase I: combination therapy with the cytotoxic agents cisplatin, Taxol, and gemcitabine. *Cancer Res* **59**: 4919–4926

Tomayko MM and Reynolds CP (1989) Determination of subcutaneous tumor size in athymic (nude) mice. *Cancer Chemother Pharmacol* **24**: 148–154

Vogt A, Qian Y, McGuire TF, Hamilton AD and Sebti SM (1996) Protein geranylgeranylation, not farnesylation, is required for the G1 to S phase transition in mouse fibroblasts. *Oncogene* **13**: 1991–1999

Zohn IM, Campbell SL, Khosravi-Far R, Rossman KL and Der CJ (1998) Rho family proteins and ras transformation: the RHOad less traveled gets congested. *Oncogene* **17**: 1415–1438

Zujewski J, Horak ID, Bol CJ, Woestenborghs R, Bowden C, End DW, Piotrovsky VK, Chiao J, Belly RT, Todd A, Kopp WC, Kohler DR, Chow C, Noone M, Hakim FT, Larkin G, Gress RE, Nussenblatt RB, Kremer AB and Cowan KH (2000) Phase I and pharmacokinetic study of farnesyl protein transferase inhibitor R115777 in advanced cancer. *J Clin Oncol* **18**: 927–941

© 2001 Cancer Research Campaign
